# *C9orf72*, age at onset, and ancestry help discriminate behavioral from language variants in FTLD cohorts

**DOI:** 10.1212/WNL.0000000000010914

**Published:** 2020-12-15

**Authors:** Beatrice Costa, Claudia Manzoni, Manuel Bernal-Quiros, Demis A. Kia, Miquel Aguilar, Ignacio Alvarez, Victoria Alvarez, Ole Andreassen, Maria Anfossi, Silvia Bagnoli, Luisa Benussi, Livia Bernardi, Giuliano Binetti, Daniel Blackburn, Mercè Boada, Barbara Borroni, Lucy Bowns, Geir Bråthen, Amalia C. Bruni, Huei-Hsin Chiang, Jordi Clarimon, Shuna Colville, Maria E. Conidi, Tom E. Cope, Carlos Cruchaga, Chiara Cupidi, Maria Elena Di Battista, Janine Diehl-Schmid, Monica Diez-Fairen, Oriol Dols-Icardo, Elisabetta Durante, Dušan Flisar, Francesca Frangipane, Daniela Galimberti, Maura Gallo, Maurizio Gallucci, Roberta Ghidoni, Caroline Graff, Jordan H. Grafman, Murray Grossman, John Hardy, Isabel Hernández, Guy J.T. Holloway, Edward D. Huey, Ignacio Illán-Gala, Anna Karydas, Behzad Khoshnood, Milica G. Kramberger, Mark Kristiansen, Patrick A. Lewis, Alberto Lleó, Gaganjit K. Madhan, Raffaele Maletta, Aleš Maver, Manuel Menendez-Gonzalez, Graziella Milan, Bruce Miller, Merel O. Mol, Parastoo Momeni, Sonia Moreno-Grau, Chris M. Morris, Benedetta Nacmias, Christer Nilsson, Valeria Novelli, Linn Öijerstedt, Alessandro Padovani, Suvankar Pal, Yasmin Panchbhaya, Pau Pastor, Borut Peterlin, Irene Piaceri, Stuart Pickering-Brown, Yolande A.L. Pijnenburg, Annibale A. Puca, Innocenzo Rainero, Antonella Rendina, Anna M.T. Richardson, Ekaterina Rogaeva, Boris Rogelj, Sara Rollinson, Giacomina Rossi, Carola Rossmeier, James B. Rowe, Elisa Rubino, Agustín Ruiz, Raquel Sanchez-Valle, Sigrid B. Sando, Alexander F. Santillo, Jennifer Saxon, Elio Scarpini, Maria Serpente, Nicoletta Smirne, Sandro Sorbi, EunRan Suh, Fabrizio Tagliavini, Jennifer C. Thompson, John Q. Trojanowski, Vivianna M. Van Deerlin, Julie Van der Zee, Christine Van Broeckhoven, Jeroen van Rooij, John C. Van Swieten, Arianna Veronesi, Emilia Vitale, Maria L. Waldö, Cathy Woodward, Jennifer Yokoyama, Valentina Escott-Price, James M. Polke, Raffaele Ferrari, for the International FTD-Genetics Consortium

**Affiliations:** From the Institute of Neurology (B.C., D.A.K., J.H., P.A.L., R.F.), School of Pharmacy (C.M.), and UCL Movement Disorders Centre (J.H.), University College London; School of Pharmacy (C.M., P.A.L.), University of Reading, Whiteknights; Neurogenetics Laboratory (M.B.-Q., C.W., J.M.P.), National Hospital for Neurology and Neurosurgery, London, UK; Aptima Clinic (Miquel Aguilar), Terrassa; Memory Disorders Unit, Department of Neurology (I.A., M.D.-F., P.P.), University Hospital Mutua de Terrassa, Barcelona; Hospital Universitario Central de Asturias (V.A., M.M.-G.), Oviedo, Spain; NORMENT (O.A.), Institute of Clinical Medicine, University of Oslo, Norway; Regional Neurogenetic Centre (Maria Anfossi, Livia Bernardi, A.C.B., M.E.C., Chiara Cupidi, F.F., Maura Gallo, R.M., N.S.), ASPCZ, Lamezia Terme; Department of Neuroscience, Psychology, Drug Research and Child Health (S.B., B.N., I.P., S.S.), University of Florence; Molecular Markers Laboratory (Luisa Benussi, Giuliano Binetti, R.G.), IRCCS Istituto Centro San Giovanni di Dio Fatebenefratelli, Brescia, Italy; Sheffield Institute for Translational Neuroscience (SITraN), Department of Neuroscience (D.B.), University of Sheffield, UK; Research Center and Memory Clinic (M.B., I.H., S.M.-G., Agustín Ruiz), Fundació ACE, Institut Català de Neurociències Aplicades, Universitat Internacional de Catalunya (UIC), Barcelona, Spain; Centre for Neurodegenerative Disorders (B.B., A.P.), Department of Clinical and Experimental Sciences, University of Brescia, Italy; Department of Clinical Neurosciences (Lucy Bowns, T.E.C., J.B.R.), Cambridge University, UK; Department of Neurology (Geir Bråthen, S.B.S.), University Hospital of Trondheim, Norway; Dept NVS, Division of Neurogeriatrics (H.-H.C., C.G., B.K., L.Ö.), Karolinska Institutet, Bioclinicum Solna, Sweden; Department of Neurology (J.C., O.D.-I., I.I.-G., A.L.), IIB Sant Pau, Hospital de la Santa Creu i Sant Pau, Universitat Autònoma de Barcelona, Spain; Anne Rowling Regenerative Neurology Clinic (S.C., G.J.T.H., S.P.) and Centre for Clinical Brain Sciences (S.P.), University of Edinburgh, UK; NeuroGenomics and Informatics, Department of Psychiatry (Carlos Cruchaga), Washington University, St. Louis, MO; Cognitive Impairment Center (M.E.D.B., Maurizio Gallucci) and Immunohematology and Transfusional Medicine Service (E.D., A.V.), Local Health Authority n.2 Marca Trevigiana, Treviso, Italy; Department of Psychiatry and Psychotherapy (J.D.-S., C.R.), School of Medicine, Technical University of Munich, Germany; Department of Neurology (D.F., M.G.K.) and Clinical Institute of Medical Genetics (A.M., B.P.), University Medical Center Ljubljana, Slovenia; Dino Ferrari Center (D.G., Elio Scarpini, M.S.), University of Milan, Italy; Cognitive Neuroscience Lab, Think and Speak Lab (J.H.G.), Shirley Ryan Ability Lab, Chicago, IL; Department of Pathology and Laboratory Medicine (Murray Grossman, EunRan Suh, J.Q.T., V.M.V.D.), Center for Neurodegenerative Diseases, Perelman School of Medicine at the University of Pennsylvania, Philadelphia; UCL Dementia Research Institute (J.H.), London; Reta Lila Weston Institute (J.H.), UCL Queen Square Institute of Neurology, UK; Institute for Advanced Study (J.H.), The Hong Kong University of Science and Technology, China; Royal Edinburgh Hospital (G.J.T.H.), UK; Taub Institute for Research on Alzheimer's Disease and the Aging Brain (E.D.H.), Columbia University, New York, NY; Department of Neurology, Memory and Aging Center (A.K., B.M., J.Y.), University of California, San Francisco; UCL Genomics (M.K., G.K.M., Y.P.), UCL Great Ormond Street Institute of Child Health, London, UK; Geriatric Center Frullone ASL Napoli 1 Centro (G.M.), Napoli, Italy; Department of Neurology (M.O.M., J.v.R., J.C.V.S.), Erasmus Medical Center, Rotterdam, the Netherlands; Rona Holdings (P.M.), Silicon Valley, CA; Newcastle Brain Tissue Resource, Institute of Neuroscience (C.M.M.), Newcastle University, Campus for Ageing and Vitality, Newcastle upon Tyne, UK; Department of Neurology (C.N.), Skåne University Hospital, Malmö, Sweden; Fondazione Policlinico Universitario A. Gemelli IRCCS (V.N.), Rome, Italy; Division of Neuroscience & Experimental Psychology (S.P.-B., A.M.T.R., S.R., J.C.T.), University of Manchester, UK; Amsterdam University Medical Center (Y.A.L.P.), VU University Medical Center, the Netherlands; Cardiovascular Research Unit (A.A.P.), IRCCS Multimedica, Milan; Neurology I, Department of Neuroscience (I.R., Elisa Rubino), University of Torino; NeurOMICS laboratory (G.M., Antonella Rendina, E.V.), Institute of Biochemistry and Cell Biology (IBBC), CNR Napoli, Italy; Manchester Centre for Clinical Neurosciences (A.M.T.R., J.S., J.C.T.), Salford Royal NHS Trust, Manchester, UK; Tanz Centre for Research in Neurodegenerative Diseases (Ekaterina Rogaeva), University of Toronto, Canada; Department of Biotechnology (B.R.), Jožef Stefan Institute, Ljubljana, Slovenia; Division of Neurology V and Neuropathology (G.R., F.T.), Fondazione IRCCS Istituto Neurologico Carlo Besta, Milan, Italy; Alzheimer's Disease and Other Cognitive Disorders Unit (R.S.-V.), Hospital Clínic of Barcelona, Spain; Clinical Memory Research Unit, Department of Clinical Sciences Malmö (C.N., A.F.S.), and Division of Clinical Sciences Helsingborg, Department of Clinical Sciences Lund (M.L.W.), Lund University, Sweden; Neurodegenerative Brain Diseases Group (J.V.d.Z., C.V.B.), Center for Molecular Neurology, VIB, Antwerp, Belgium; Medical Research Council Centre for Neuropsychiatric Genetics and Genomics (V.E.-P.), Division of Psychological Medicine and Clinical Neurosciences and Dementia Research Institute, Cardiff University, UK; Instituto de Investigación Sanitaria del Principado de Asturias (V.A.), Oviedo, Asturias; Fundació per la Recerca Biomèdica i Social Mútua Terrassa (I.A., M.D.-F., P.P.), Barcelona; Centro de Investigación Biomédica en Red de Enfermedades Neurodegenerativas (CIBERNED) (M.B., J.C., O.D.-I., I.H., I.I.-G., A.L., S.M.-G., Agustín Ruiz), Instituto de Salud Carlos III, Madrid, Spain; MRC Cognition and Brain Sciences Unit (Lucy Bowns, T.E.C., J.B.R.), Cambridge University, UK; Department of Neuromedicine and Movement Science (Geir Bråthen, S.B.S.), Norwegian University of Science and Technology, Trondheim, Norway; Unit for Hereditary Dementias (H.-H.C., C.G., B.K., L.Ö.), Theme Aging, Karolinska University Hospital, Solna, Sweden; Medical Faculty (D.F., M.G.K.), University of Ljubljana, Slovenia; Fondazione IRCCS Ca'Granda (D.G., Elio Scarpini, M.S.), Ospedale Policlinico, Milan, Italy; Penn Center for Frontotemporal Degeneration (Murray Grossman), Philadelphia, PA; Universidad de Oviedo (M.M.-G.), Asturias, Spain; IRCCS Fondazione Don Carlo Gnocchi (B.N., S.S.), Florence; Istituto di Medicina Genomica (V.N.), Università Cattolica del sacro Cuore, Rome, Italy; Amsterdam Neuroscience (Y.A.L.P.), the Netherlands; Department of Medicine and Surgery (A.A.P.), University of Salerno, Baronissi (SA), Italy; Faculty of Chemistry and Chemical Technology (B.R.), University of Ljubljana, Slovenia; Institud d'Investigacions Biomèdiques August Pi i Sunyer (R.S.-V.), Barcelona, Spain; Department of Biomedical Sciences (J.V.d.Z., C.V.B.), University of Antwerp, Belgium; and Department of Comparative Biomedical Sciences (P.A.L.), The Royal Veterinary College, London, UK.

## Abstract

**Objective:**

We sought to characterize *C9orf72* expansions in relation to genetic ancestry and age at onset (AAO) and to use these measures to discriminate the behavioral from the language variant syndrome in a large pan-European cohort of frontotemporal lobar degeneration (FTLD) cases.

**Methods:**

We evaluated expansions frequency in the entire cohort (n = 1,396; behavioral variant frontotemporal dementia [bvFTD] [n = 800], primary progressive aphasia [PPA] [n = 495], and FTLD–motor neuron disease [MND] [n = 101]). We then focused on the bvFTD and PPA cases and tested for association between expansion status, syndromes, genetic ancestry, and AAO applying statistical tests comprising Fisher exact tests, analysis of variance with Tukey post hoc tests, and logistic and nonlinear mixed-effects model regressions.

**Results:**

We found *C9orf72* pathogenic expansions in 4% of all cases (56/1,396). Expansion carriers differently distributed across syndromes: 12/101 FTLD-MND (11.9%), 40/800 bvFTD (5%), and 4/495 PPA (0.8%). While addressing population substructure through principal components analysis (PCA), we defined 2 patients groups with Central/Northern (n = 873) and Southern European (n = 523) ancestry. The proportion of expansion carriers was significantly higher in bvFTD compared to PPA (5% vs 0.8% [*p* = 2.17 × 10^−5^; odds ratio (OR) 6.4; confidence interval (CI) 2.31–24.99]), as well as in individuals with Central/Northern European compared to Southern European ancestry (4.4% vs 1.8% [*p* = 1.1 × 10^−2^; OR 2.5; CI 1.17–5.99]). Pathogenic expansions and Central/Northern European ancestry independently and inversely correlated with AAO. Our prediction model (based on expansions status, genetic ancestry, and AAO) predicted a diagnosis of bvFTD with 64% accuracy.

**Conclusions:**

Our results indicate correlation between pathogenic *C9orf72* expansions, AAO, PCA-based Central/Northern European ancestry, and a diagnosis of bvFTD, implying complex genetic risk architectures differently underpinning the behavioral and language variant syndromes.

Frontotemporal lobar degeneration (FTLD) refers to the second most common form of young-onset dementia after Alzheimer disease.^1^ The major clinical syndromes are behavioral variant frontotemporal dementia (bvFTD)^2^ or language dysfunctions, broadly called primary progressive aphasia (PPA); the latter is subdivided into semantic dementia or semantic variant PPA and progressive nonfluent aphasia (PNFA) or nonfluent/agrammatic variant PPA.^2,3^ FTLD can also occur together with motor neuron disease (MND) or amyotrophic lateral sclerosis in a continuous spectrum of phenotypes.^4^

In FTLD, repeat expansions in *C9orf72*^5^ have been previously reported to occur in ∼25%^6–10^ of familial and ∼6%^11^ of sporadic cases (i.e., individuals with no clear familial history or genetic aetiology^12^). Several studies had shown high frequencies of pathogenic *C9orf72* expansions in Northern vs Southern European patients (North–South axis), especially in historically isolated populations (such as the Finnish^13,14^), leading to the hypothesis that a Scandinavian founder might be at the basis of the spread of the *C9orf72* expansion.^15^ Other studies (based on the geographic location of the recruiting sites) challenged the North–South axis concept, reporting a high frequency (∼25%) of pathogenic expansions in the Spanish population^10^ or implying to the existence of more than 1 risk haplotype.^16–19^

Patients with FTLD with abnormal *C9orf72* repeat expansions exhibit marked phenotypic and pathologic heterogeneity, suggesting presence of additional (genetic and environmental) modifiers.^20^ Despite conflicting studies reporting either direct or inverse correlation between repeat length and age at onset (AAO), *C9orf72* expansions have been suggested to act as a genetic modifier of AAO.^16,21–24^

We analyzed 1,396 FTLD cases gathered through the International FTD Genetics Consortium (IFGC) (ifgcsite.wordpress.com/) phase III initiative, aiming at (1) characterizing *C9orf72* expansions in relation to genetic ancestry and AAO and (2) assessing the usefulness of these measures in discriminating the behavioral from the language variant syndrome.

## Methods

### Standard protocol approvals, registrations, and patient consents

Each contributing site obtained written informed consent from all patients to be part of extended genetic studies; the current study is approved under institutional review board approval 9811/001.

### Cohort, clinical phenotyping

FTLD cases were collected between 2016 and 2018 (within the IFGC phase III project [ifgcsite.wordpress.com/ongoing-projects/]). The samples were recruited by clinicians and research groups who are part of the IFGC network and based in Italy, Spain, Germany, the Netherlands, Belgium, the United Kingdom, Sweden, Norway, Slovenia, or the United States (supplementary table 1, doi.org/10.5522/04/12418157). Patients were diagnosed at each contributing site (supplementary table 2, doi.org/10.5522/04/12418157) in a harmonized fashion according to international consensus criteria such as those of Neary et al.^2^ (for FTLD), Rascovsky et al.^25^ (for bvFTD), Gorno-Tempini et al.^3^ (for PPA [semantic dementia or PNFA]), and Strong et al.^4^ (for FTLD-MND).

### Genotyping, *C9orf72* repeat expansions, and analysis cohorts

**Figure 1:**
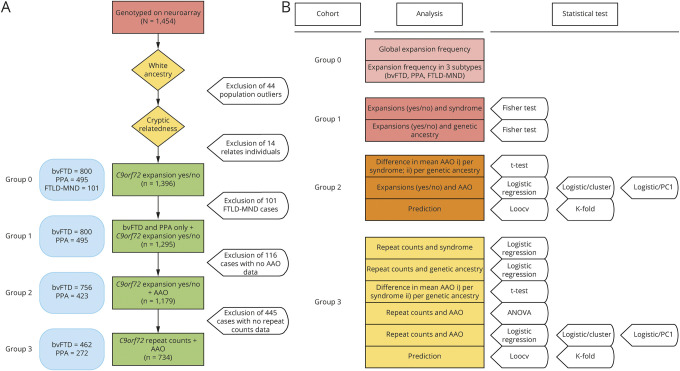
Cohorts and analysis workflow (A) Cohorts. (B) Analysis workflow. K-fold regression model. AAO = age at onset; ANOVA = analysis of variance; bvFTD = behavioral variant frontotemporal dementia; FTLD = frontotemporal lobar degeneration; logistic/cluster = logistic regression using cluster as covariate; logistic/PC1 = logistic regression using PC1 as covariate; LOOCV = leave-one-out cross-validation regression model; MND = motor neuron disease; PPA = primary progressive aphasia.

A total of 1,454 cases were successfully genotyped by means of the NeuroArray^26^ on the Illumina Infinium platform. Genotypes were used to inform on population substructure via standard principal components analysis (PCA) (supplementary figure 1, doi.org/10.5522/04/12418157), which led to the exclusion of 44 population outliers, and allowed us to address population substructure within the cohort (we identified 2 distinct [Nordic and Mediterranean] clusters; supplementary figure 2, doi.org/10.5522/04/12418157). We also assessed cryptic relatedness and excluded 14 first- or second-degree related individuals, leaving a cohort of 1,396 cases (group 0)—for which *C9orf72* expansion status (i.e., presence/absence of pathogenic expansions) was known—for analyses. Frequencies of pathogenic expansions were assessed in group 0 and further analyses were performed in (1) 1,295 cases (group 1: n = 800 bvFTD and n = 495 PPA) with known *C9orf72* expansion status; (2) 1,179 cases (group 2; n = 756 bvFTD and n = 423 PPA) with known *C9orf72* expansion status and AAO data available; and (3) 734 cases (group 3; n = 462 bvFTD and n = 272 PPA) with AAO and repeat counts (rc; screened via repeat-primed PCR) (see references 27 and 28; supplementary Methods and supplementary figure 3, doi.org/10.5522/04/12418157; and figure 1A).

### Statistical analyses

We first assessed the frequency of pathogenic expansions in the entire cohort (group 0). The information on presence/absence of expansions was used as a binary variable (0 = absence of expansion; 1 = presence of expansion). We then investigated differences in the frequencies of pathogenic expansions across bvFTD and PPA and the Nordic and Mediterranean clusters in group 1 (Fisher exact test) and in group 3 (logistic regression); in the latter, we used rc as a categorical variable (using no, short, intermediate, and long as factor levels) considering the following 4 categories: no expansions (rc = 2/3), short expansions (4 ≤ rc ≤ 8), intermediate expansions (9 ≤ rc ≤ 24), and long expansions (rc ≥ 25), the latter representing expansions in the pathogenic range (see references 10 and 22, supplementary Methods, and supplementary figure 3, doi.org/10.5522/04/12418157).

We then evaluated association between AAO and syndrome, genetic ancestry, and expansions (i.e., presence/absence used as a binary variable; see above) alone and with genetic ancestry as a covariate in group 2 (*t* test and logistic regression) and in group 3 (*t* test, analysis of variance with Tukey post hoc test, and logistic and linear mixed-effects model). In the latter case, we used rc as a categorical variable (see above).

Finally, we sought to build a model to predict syndrome (bvFTD vs PPA) using (1) presence/absence of pathogenic expansions (as binary variable [see above] for group 2) or (2) rc (as categorical variable [see above] for group 3), ancestry as binary variable, and AAO as continuous variable using logistic regression models (i.e., the leave-one-out cross-validation [LOOCV] and the K-fold models). A summary of the analyses workflow can be found in figure 1B.

All analyses were performed using R studio (version 3.6.0, studio version 1.2.1335).

### *C9orf72* locus risk haplotype

Twenty (rs1110264, rs1110155, rs2150336, rs1161680, rs2589054, rs1822723, rs4879515, rs895023, rs868856, rs1977661, rs903603, rs12349820, rs10122902, rs2282241, rs1948522, rs1982915, rs2453556, rs702231, rs696826, and rs247751) of the original 42 single nucleotide polymorphisms (SNPs) constituting the (Finnish) risk haplotype^29^ were available on the NeuroArray.^26^ We filtered out 7 markers in order to keep 13 informative SNPs (rs1822723, rs4879515, rs868856, rs1977661, rs903603, rs10122902, rs2282241, rs1948522, rs1982915, rs2453556, rs702231, rs696826, and rs2477518) matching 13 of the 20 used in Mok et al.^15^ We evaluated the proportion of cases carrying at least 1 risk allele (as in Mok et al.^15^) for each marker assessing expansion vs nonexpansion carriers (with/without ancestry stratification).

### Data availability

All data generated or analyzed during this study are included in this published article and supplementary files 1 and 2 at doi.org/10.5522/04/12418157.

## Results

### *C9orf72* expansions frequency and syndromes

We assessed the frequency of pathogenic expansions in the entire cohort and across the different syndromes in the group 0 cases (figure 1). Four percent of all cases (56/1,396 [4%]) carried pathogenic expansions. These were most frequent in FTLD-MND (12/101 [11.9%]) followed by bvFTD (40/800 [5%]) and PPA (4/495 [0.8%]). The higher prevalence of pathogenic expansions in bvFTD vs PPA was statistically significant (Fisher exact test: *p* = 2.17 × 10^−5^; OR 6.4; 95% CI 2.31–24.99, table 1). We further explored this finding in the group 3 cases using logistic regression to assess association between expansion length (represented by 4 rc factor levels: short, intermediate, and long expansions, tested against no expansions) and syndromes (bvFTD vs PPA). Expansion length discriminated bvFTD from PPA with a trend that was significant in the intermediate (*p* = 4.7 × 10^−2^; OR 1.6; CI 0.0061 [2.5%]–0.94 [97.5%]) and long (*p* = 1.9 × 10^−3^; OR 7.2; CI 0.86 [2.5%]–3.45 [97.5%]) rc ranges (with a ∼90% probability of a bvFTD diagnosis supported by the latter; supplementary table 3, doi.org/10.5522/04/12418157).

**Table 1:**
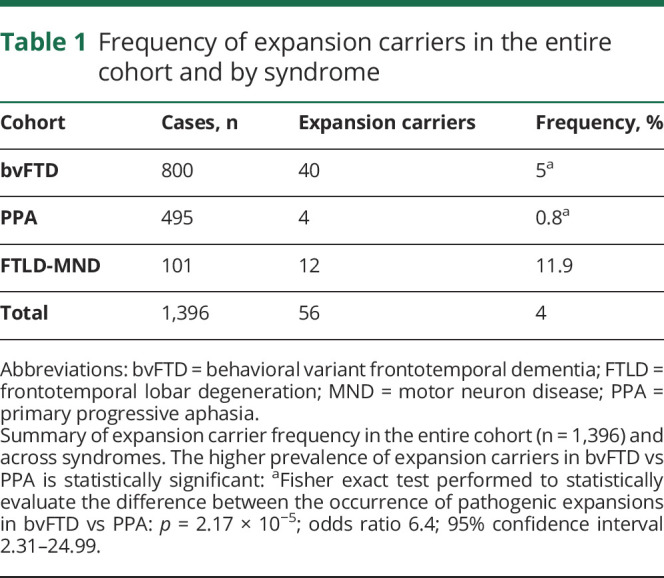
Frequency of expansion carriers in the entire cohort and by syndrome

### *C9orf72* expansions (and rc) and genetic ancestry

We performed PCA (PC1 vs PC2, supplementary figure 2A; PC1 vs PC3, supplementary figure 2B, doi.org/10.5522/04/12418157) to cluster the group 1 cases based on their genetic makeup. There were 2 major clusters: cluster 1 (Mediterranean) included most of the cases (439/500 [87.8%]) recruited from Southern European sites (Italy and Spain); cluster 2 (Nordic) included most of the cases (627/795 [78.8%]) recruited from Central and Northern European sites (Belgium, the Netherlands, Germany, the United Kingdom, Norway, and Sweden). Samples recruited from Eastern European (Slovenia) and North American sites distributed across both clusters, although with a higher prevalence within cluster 2 (167/795 [21%]) vs cluster 1 (42/500 [8.4%]).

We observed a significantly higher prevalence of pathogenic expansions in the Nordic (35/795 [4.4%]) vs the Mediterranean (9/500 [1.8%]) cluster (Fisher exact test: *p* = 1.1 × 10^−2^; OR 2.5; CI 1.17–5.99, table 2). We further evaluated this finding in the group 3 cases using logistic regression to assess association between expansion length (see above) and genetic ancestry. Expansion length discriminated the Nordic from Mediterranean cluster with a trend that was significant in the intermediate (*p* = 9.7 × 10^−4^, OR 2.2; CI 0.32 [2.5%]–1.25 [97.5%]) and long (*p* = 4.7 × 10^−4^, OR 9.3; CI 1.12 [2.5%]–3.7 [97.5%]) rc ranges (with a ∼90% probability of Nordic ancestry supported by the latter; supplementary table 4, doi.org/10.5522/04/12418157).

**Table 2:**
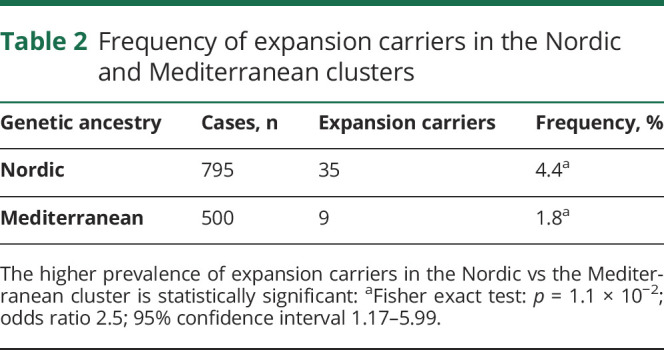
Frequency of expansion carriers in the Nordic and Mediterranean clusters

Provided differences in syndromes prevalence and distribution across the Nordic and Mediterranean clusters—bvFTD (469/795 [59%] vs 331/500 [66.2%]) and PPA (326/795 [41%] vs 169/500 [33.8%]), respectively (supplementary table 5, doi.org/10.5522/04/12418157)—we analyzed the distribution of pathogenic expansions across syndromes and clusters. Stratified Fisher exact test showed significant differences in the distribution of the pathogenic expansions between bvFTD and PPA in the Nordic (but not the Mediterranean) cluster (*p* = 1 × 10^−4^; OR 7.87; 95% CI 2.43–40.52), and between the Nordic and the Mediterranean clusters for the bvFTD (but not PPA) syndrome (*p* = 1.9 × 10^−2^; OR 2.95; 95% CI 1.31–7.52), suggesting that ancestry (Nordic) and syndrome (bvFTD) are independently associated with pathogenic expansions (table 3).

**Table 3:**
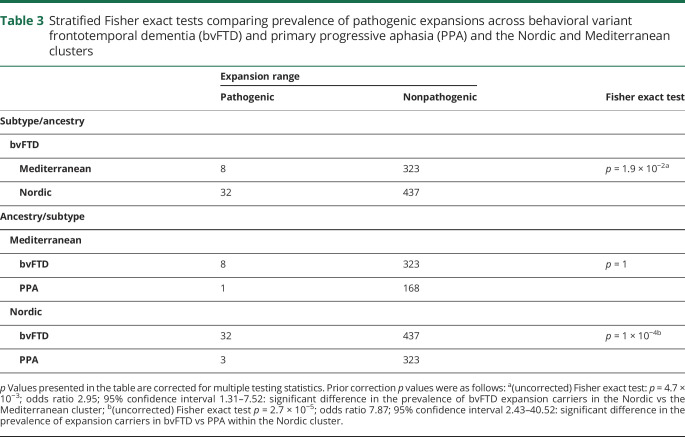
Stratified Fisher exact tests comparing prevalence of pathogenic expansions across behavioral variant frontotemporal dementia (bvFTD) and primary progressive aphasia (PPA) and the Nordic and Mediterranean clusters

### *C9orf72* repeat expansions (and counts [rc]) and AAO

**Figure 2:**
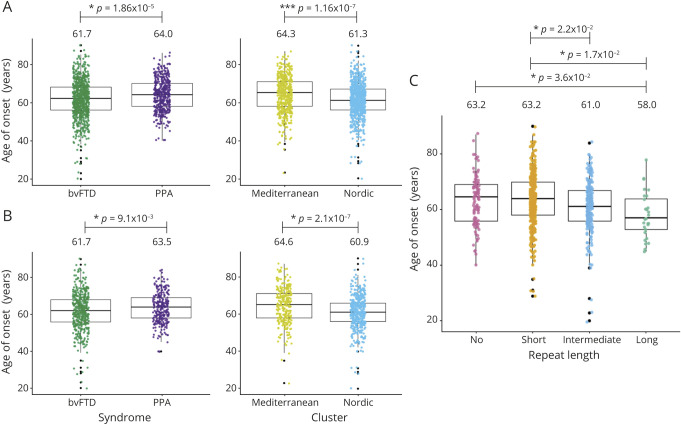
Association between age at onset (AAO) and ancestry, syndrome, and expansion length (A) AAO in the group 2 cases. Mean AAO behavioral variant frontotemporal dementia (bvFTD) (61.7) and primary progressive aphasia (PPA) (64) (*t* test: *p* = 1.86 × 10^−5^; confidence interval [CI] −3.34 to 1.25); mean AAO Nordic (61.3) and Mediterranean (64.3) clusters (*t* test: *p* = 1.16 × 10^−7^; CI 1.86–4.03). (B) AAO in the group 3 cases. Mean AAO bvFTD (61.7) and PPA (63.5) (*t* test: *p* = 9.1 × 10^−3^; CI −3.11 to 0.44); mean AAO Nordic (60.9) and Mediterranean (64.6) (*t* test: *p* = 2.1 × 10^−7^; CI 2.32–5.09). (C) AAO in the group 3 cases. Mean AAO for both no and short expansions (63.2), for intermediate expansions (61), and for long expansions (58) evaluated via analysis of variance test.

We assessed AAO in the group 2 cases (figure 1). Mean AAO was significantly different between the bvFTD (61.7) and PPA (64) syndromes (*t* test: *p* = 1.86 × 10^−5^; CI −3.34 to −1.25) and the Nordic (61.3) and Mediterranean (64.3) clusters (*t* test: *p* = 1.16 × 10^−7^; CI 1.86–4.03) (figure 2A and supplementary table 6, A and B, doi.org/10.5522/04/12418157). We then assessed the relationship between pathogenic expansions and AAO via logistic regression. First, we identified a significant correlation between a decrease in AAO and presence of pathogenic expansions (*p* = 7.7 × 10^−4^; *R*^2^ = 0.008; CI −8.05 [2.5%] to −2.13 [97.5%]). When we included genetic ancestry in the model, we observed a significant correlation with a decrease in AAO, with no difference in using either cluster (*p* = 2.3 × 10^−3^; CI −7.5 [2.5%] to −1.63 [97.5%] for pathogenic expansions; *p* = 2.3 × 10^−7^; CI −3.9 [2.5%] to −1.77 [97.5%] for cluster; *R*^2^ = 0.03) or PC1 (*p* = 2.1 × 10^−3^; CI −7.5 [2.5%] to −1.66 [97.5%] for pathogenic expansions; *p* = 6.4 × 10^−7^; CI 30.1 [2.5%]–68.9 [97.5%] for PC1; *R*^2^ = 0.028) as covariate and an almost 4-fold goodness of fit increase (supplementary table 7, A–C, doi.org/10.5522/04/12418157). Of note, when comparing the 2 regression models (with/without genetic ancestry as covariate) through the log-likelihood *R*^2^ ratio test, the difference (between the 2 models) appeared not to be due to chance (*p* < 10^−12^) (supplementary table 7, B and C, doi.org/10.5522/04/12418157).

We further evaluated the relationship between expansion length (represented by 4 rc factor levels—short, intermediate, and long expansions, tested against no expansions) and AAO in the group 3 cases (figure 1). First, we independently analyzed association between AAO and (1) genetic ancestry—mean AAO 60.9 and 64.6 in the Nordic and Mediterranean cluster, respectively (*t* test: *p* = 2.1 × 10^−7^; CI 2.32–5.09; supplementary table 8A, doi.org/10.5522/04/12418157); (2) syndrome—mean AAO 61.7 and 63.5 in the bvFTD and PPA syndromes, respectively (*t* test: *p* = 9.1 × 10^−3^; CI −3.11 to −0.44; supplementary table 8B, doi.org/10.5522/04/12418157), and (3) expansion length—mean AAO 63.2 for both no and short expansions, 61 for intermediate expansions, and 58 for long expansions (analysis of variance *p* = 3.6 × 10^−2^; CI −10.2 to −0.23 for long vs no expansions) (supplementary table 8D, figure 2B and C: doi.org/10.5522/04/12418157). We then assessed the relationship between expansion length (see above) and AAO via logistic regression. First, we identified a significant correlation between a decrease in AAO and both intermediate and long expansions (*p* = 4 × 10^−2^; CI −4.36 [2.5%] to −0.96 [97.5%] for intermediate and p = 7 × 10^−3^; CI −9.05 [2.5%] to −1.43 [97.5%] for long expansions; *R*^2^ = 0.017) (supplementary table 9A, doi.org/10.5522/04/12418157). When we included genetic ancestry in the model, we observed a significant correlation with a decrease in AAO, no difference in using either cluster (*p* = 4.7 × 10^−2^; CI −7.65 [2.5%] to −0.05 [97.5%] for long vs no expansion; *p* = 2.38 × 10^−6^; CI −4.73 [2.5%] to −1.97 [97.5%] for cluster; *R*^2^ = 0.045) or PC1 (*p* = 5.98 × 10^−2^; CI −7.5 [2.5%] to 0.14 [97.5%] for long vs no expansion; *p* = 1.2 × 10^−6^; CI 39.8 [2.5%] –92.9 [97.5%] for PC1; *R*^2^ = 0.047) as covariate and an almost 3-fold goodness of fit increase (supplementary table 9, A–C, doi.org/10.5522/04/12418157). Of note, when comparing the 2 regression models (with/without genetic ancestry as covariate) through the log-likelihood *R*^2^ ratio test, the difference (between the 2 models) appeared not to be due to chance (*p* < 10^−12^) (supplementary table 9, B and C, doi.org/10.5522/04/12418157). These findings were further supported by nonlinear mixed-effects model regression using genetic ancestry as random effect covariate (for long vs no expansion; see supplementary table 10, doi.org/10.5522/04/12418157).

### *C9orf72* locus risk haplotype

All of the risk alleles for the 13 markers—shortest informative stretch of the original risk haplotype^15,29^ available to us—were seen in (1) 40/56 (71.4%) expansion carriers vs 380/1,340 (28.4%) nonexpansion carriers in the entire cohort; (2) 33/47 (70.2%) expansion carriers vs 228/826 (27.6%) nonexpansion carriers in the Nordic cluster; and (3) 7/9 (77.8%) expansion carriers vs 152/514 (29.6%) nonexpansion carriers in the Mediterranean cluster. Comparing the proportion of risk allele carriers (expansion vs nonexpansion carriers) for each single marker, 5/13 markers (rs4879515, rs868856, rs903603, rs2282241, rs2453556) were significant in the Nordic cluster, and none in the Mediterranean cluster (supplementary figure 4, doi.org/10.5522/04/12418157). Rs2477518 showed variable frequencies for the risk allele (T) across expansion vs nonexpansion carriers (and the 2 clusters), thus making this most probably a negligible marker within this stretch, as hinted previously.^15,17^ Rs3849942, previously suggested as a surrogate marker for the risk haplotype,^15^ was not among the SNPs available to us. We used rs868856, displaying strongest linkage disequilibrium with rs3849942 (D' = 0.96; *R*^2^ = 0.7; ldlink.nci.nih.gov/), as informative proxy: the risk allele segregated differently across expansion vs nonexpansion carriers in the Nordic and Mediterranean cluster (as for rs2453556), possibly suggesting these 2 as the most conserved markers of the original risk haplotype across populations in expansion carriers (highlighted in blue in supplementary figure 4, doi.org/10.5522/04/12418157).

### Syndrome prediction

We then sought to build a model to predict syndrome (bvFTD vs PPA) and assess its accuracy. We analyzed both groups 2 and 3 cases using expansion status (presence/absence of expansion for group 2 and the 4 rc factor levels for group 3 [see Methods]), genetic ancestry (using either cluster or PC1) as binary variables, and AAO as a continuous variable in logistic regression models. We observed an accuracy of ∼0.64 (group 2; supplementary table 11, doi.org/10.5522/04/12418157) and ∼0.62 (group 3; supplementary table 12, doi.org/10.5522/04/12418157) in predicting bvFTD; there were no differences in the outcome when using either cluster or PC1 as covariates in both (LOOCV and K-fold) models.

## Discussion

This study aimed to characterize *C9orf72* expansions in relation to genetic ancestry and AAO and to assess the usefulness of these measures in discriminating the behavioral from the language variant syndrome in a large pan-European cohort of 1,396 FTLD cases.

To our knowledge, the current work is unique in that, prior to characterizing the expansions, we excluded population-substructure bias using genome-wide genotyping data to cluster the cases on the basis of their genetic makeup. After PCA, we identified 2 distinct clusters including samples with geographic ancestry corresponding to Southern Europe (Mediterranean cluster) and Central/Northern Europe (Nordic cluster). Our analyses not only showed that patients from the Nordic cluster presented significantly higher frequency of pathogenic *C9orf72* expansions compared to the Mediterranean cluster, but also that a core stretch of markers (n = 8) of the Finnish risk haplotype^29^ appeared to be conserved across the Nordic expansion carriers, whereas there was a similar tendency for (just) 2 of such markers in the Mediterranean expansion carriers. Several studies had shown high frequencies of long *C9orf72* expansions in Northern vs Southern European patients (North–South axis).^13–15^ Other studies (based on the geographic location of the recruiting sites) challenged the North–South axis concept,^10^ or the founder effect implying the existence of more than 1 risk haplotype.^16–19^ All this taken together, our current data appear to support the North–South axis hypothesis and suggest that rearrangements (and instability)^16,19^ at the *C9orf72* locus might have occurred, reducing the level of conservation of the original risk haplotype across the European population.

**Figure 3:**
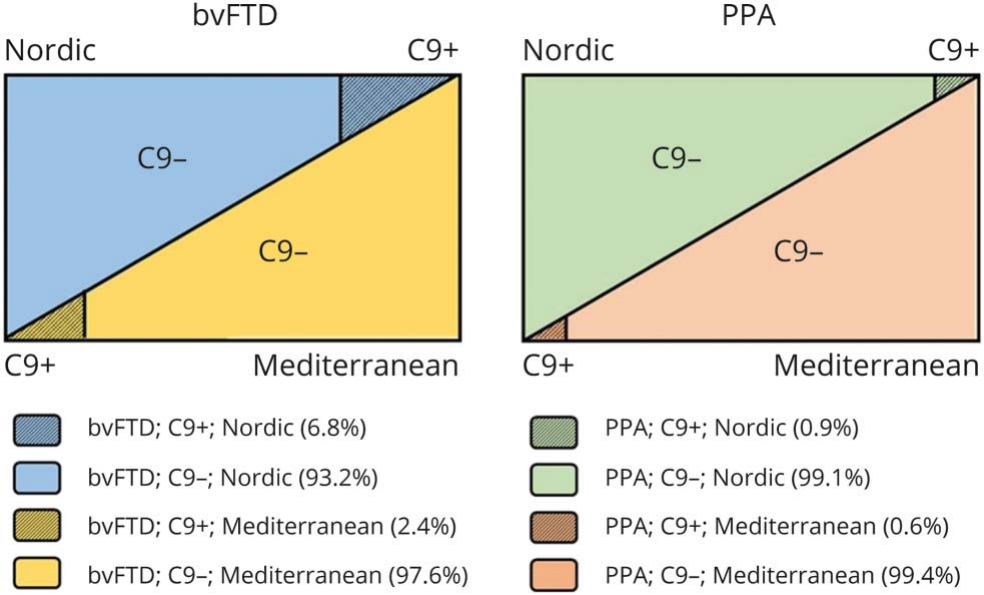
Patient subpopulations (behavioral variant frontotemporal dementia [bvFTD] and primary progressive aphasia [PPA] syndromes) based on *C9orf72* expansion genetic signatures and ancestry

We found pathogenic expansions in ∼4% of all cases and that the proportion of expansion carriers was significantly higher in bvFTD compared to PPA. The fact that we overall identified significant association between pathogenic expansions and a diagnosis of bvFTD and Central/Northern European ancestry—findings in line with previous reports^8,10,13,20,30–34^—suggests that *C9orf72* expansions might serve as useful genetic fingerprint to define subpopulations of FTLD (figure 3). Of note, we observed a trend of association with syndrome (bvFTD) and genetic ancestry (Central/Northern European) already supported by the intermediate repeat counts (9 ≤ rc ≤ 24) category. This appears in line with previous reports suggesting that individuals with 7–24 alleles might have an increased risk to convert to carriers of pathologic repeat expansions^10,22^ and may, altogether, be useful information in the context of diagnostics.

Despite some previous conflicting reports of direct (or inverse) correlation between *C9orf72* expansions and AAO,^16,21,23^ we (as others^22,24^) found a significant inverse correlation between *C9orf72* expansion length and AAO. In addition, and interestingly, our data also indicate that Central/Northern European genetic ancestry contributes to a decreased AAO (independently from the expansions), possibly implying a more complex genetic signature (or architecture), and subsequently molecular mechanisms, underpinning this feature. Clearly, disease mechanisms that involve *C9orf72* expansion length and AAO are complex, thus it is likely that additional factors might further modulate their relationship and effect on the phenotype (see also Babić Leko et al.^5^).

While using expansion length, genetic ancestry, and AAO in a regression model to discriminate behavioral from language variant subtypes, we found that such measures supported a prediction of bvFTD with 64% accuracy.

Our results have a number of implications. First, provided that significant variation exists in the genetic architecture of the Caucasian population,^35^ genetic variability characterizing and differentiating Nordic vs Mediterranean subjects (such as in the case of our cohort) might influence predisposition to harboring longer repeat expansions. In other repeat expansion diseases—e.g., Huntington disease or other microsatellite diseases, including myotonic dystrophy and spinocerebellar ataxias^35^—the presence of specific haplogroups in Western European populations occurs with a manifold increase in prevalence of repeats compared to other ethnic groups and populations.^36^ Second, different genetic risk architectures underpinning different (and possibly genetically more homogeneous) subpopulations of patients may exist within the FTLD population.

In a nutshell, our results imply that a significantly higher proportion of FTLD cases, with Nordic rather than Mediterranean genetic ancestry, is likely to develop bvFTD in presence of intermediate and long (pathogenic) expansions, whereas long (pathogenic) expansions are (almost) negligible in PPA, regardless of ancestry. Clearly, multiple factors including genetic heterogeneity, epigenetic changes, ethnicity, as well as environmental factors and habits that may subsist within and across multicultural cohorts, all together, contribute to disease predisposition, onset, and progression.^22,37,38^ These concepts, reinforced by our study, warrant further characterization of genetic, environmental, and additional clinical measures to fine-tune models able to predict disease outcome to complement diagnostic criteria, and possibly assist in the identification of informative cohorts for tailored clinical trials and the development of effective personalized therapies.

## Glossary

AAO: age at onset
bvFTD: behavioral variant frontotemporal dementia
FTLD: frontotemporal lobar degeneration
IFGC: International FTD Genetics Consortium
LOOCV: leave-one-out cross-validation
MND: motor neuron disease
PCA: principal components analysis
PNFA: progressive nonfluent aphasia
PPA: primary progressive aphasia
rc: repeat counts
SNP: single nucleotide polymorphism
